# Diet optimization using linear programming to develop low cost cancer prevention food plan for selected adults in Kuala Lumpur, Malaysia

**DOI:** 10.1186/s12889-019-6872-4

**Published:** 2019-06-13

**Authors:** Reham Alaini, Roslee Rajikan, Siti Masitah Elias

**Affiliations:** 1 Dietetics Programme, Faculty of Health Sciences, Universiti Kebangsaan Malaysia, 50300 Kuala Lumpur, Malaysia; 2 Financial Mathematics Programme, Faculty of Science and Technology, Universiti Sains Islam Malaysia, 71800 Nilai, Negeri Sembilan Malaysia

**Keywords:** Balanced diet, Cancer prevention, Linear programming

## Abstract

**Background:**

Poor dietary habits have been identified as one of the cancer risks factors in various epidemiological studies. Consumption of healthy and balance diet is crucial to reduce cancer risk. Cancer prevention food plan should consist of all the right amounts of macronutrients and micronutrients. Although dietary habits could be changed, affordability of healthy foods has been a major concern, as the price of healthy foods are more expensive the unhealthy counterparts.

**Methods:**

Therefore, using linear programming, this study is aimed to develop a healthy and balanced menu with minimal cost in accordance to individual needs that could in return help to prevent cancer. A cross sectional study involving 100 adults from a local university in Kuala Lumpur was conducted in 3 phases. The first phase is the data collection for the subjects, which includes their socio demographic, anthropometry and diet recall. The second phase was the creation of a balanced diet model at a minimum cost. The third and final phase was the finalization of the cancer prevention menu. Optimal and balanced menus were produced based on respective guidelines of WCRF/AICR (World Cancer Research Fund/ American Institute for Cancer Research) 2007, MDG (Malaysian Dietary Guidelines) 2010 and RNI (Recommended Nutrient Intake) 2017, with minimum cost.

**Results:**

Based on the diet recall, most of subjects did not achieve the recommended micronutrient intake for fiber, calcium, potassium, iron, B12, folate, vitamin A, vitamin E, vitamin K, and beta-carotene. While, the intake of sugar (51 ± 19.8 g), (13% ± 2%) and sodium (2585 ± 544 g) was more than recommended. From the optimization model, three menus, which met the dietary guidelines for cancer prevention by WCRF/AICR 2007, MDG 2010 and RNI 2017, with minimum cost of RM7.8, RM9.2 and RM9.7 per day were created.

**Conclusion:**

Linear programming can be used to translate nutritional requirements based on selected Dietary Guidelines to achieve a healthy, well-balanced menu for cancer prevention at minimal cost. Furthermore, the models could help to shape consumer food choice decision to prevent cancer especially for those in low income group where high cost for health food has been the main deterrent for healthy eating.

## Background

A balanced diet can be categorized with the concept of diversity and simplicity that is related with the perception of healthy eating [1]. Healthy diets are obtained by taking foods that contain recommended dose of macronutrients and micronutrients [2]. Dietary Guidelines can be adapted to promote health and preventing diet-related chronic diseases including cardiovascular disease, type 2 diabetes, some cancers, and obesity [3]. Throughout recorded history, wise choices of food and drink and of habitual behavior have been recommended to protect against cancer as well as other diseases, and to improve wellbeing. In Malaysia cancer is one of the major health problems [4]. It is undeniably one of the most important non-communicable diseases in Malaysia and contributed to 13.56% of all deaths occurred in the Ministry of Health Hospitals in 2015 [5]. Nutrition and related factors such as physical activity, obesity believed to contribute crucially to cancer occurrence [6].

Socioeconomic status of the population plays a critical role in eating patterns and food choices. Several studies have shown that energy-dense foods are foods that are commonly chosen by the low socioeconomic class due to their cheaper price [7] as financial resources are limited [8–11]. Foods of lower nutritional value and lower-quality diets generally cost less per calorie and tended to be selected by groups of lower socioeconomic status. A number of nutrient-dense foods were available at low cost but were not always palatable or culturally acceptable to the low-income consumer [12]. Furthermore, high price of healthy foods has been one of the main deterrents for healthy eating among the lower income group [13–15]. The encouragement toward healthy, balanced, affordable and palatable diet among the low-income group may improve their overall health status and eventually reduce the prevalence of chronic diseases in Malaysia including cancer.

Linear programming can be used to formulate minimum cost menus while making sure it met all the criteria of all macronutrients and micronutrients that has been set by dietary guidelines [16]. It is used in diet problem-solving techniques by creating a model that contains all the optimal food, cost and quality of a diet. By using linear programming, the majority of populations can benefit from an optimal diet at a minimum cost, which enables them to have an adequate daily nutrition within their financial potentials.

Linear programming has been applied in the Pacific Northwest of the USA, which was the only study that presents an application of mathematical optimization tools of dietary guidelines for cancer prevention. Six-specific food plans were generated that met both the key 2007 dietary recommendations for cancer prevention issued by the WCRF/AICR 2007 and the DRIs set by the Institute of Medicine [17].

Currently there is no study done in Malaysia to create a balanced, optimal diet for cancer prevention using linear programming at a minimum cost. Hence, this study aimed to construct a balanced, palatable and affordable diet that helps to prevent cancer at the lowest price possible to make it affordable and achievable for low income individuals.

## Methods

This study was approved by the National University of Malaysia Medical Research Ethics Committee (UKMREC) (NN-2017-128). One hundred healthy adults aged 19 and above both male and female from a local university in Kuala Lumpur, staff and students, were randomly selected to participate in this study. Dietary intake and socio-demographic data were collected between September–October 2017.

### Data collection

A set of questionnaires was distributed to the subjects to assess their socio-demographic profile. Basic information such as age, gender, marital status, and education level, lifestyle of the participants such as smoking habits, history of weight and height were obtained. Body mass index (BMI) was calculated by using weight and height and classified based on WHO BMI classification into underweight, normal, overweight and obesity in adults [18]. Eating pattern for both male and female staff and students of a local university in Kuala Lumpur, were recorded and assessed by three-day food records, where the subjects had to document their food intake for two days in a weekday and one over the weekend. Based on the subject’s diet history information, a food list is prepared and the price for each food items was obtained from the Ministry of Domestic Trade, Cooperative, and Consumerism (KPDNKK). The food prices were set in terms of price per serving size.

### Statistical analysis

Dietary intake was initially analyzed using Nutritionist Pro™ software version 4.0.0 and compared with Recommended Nutrient Intake (RNI) 2017 for Malaysian [19]. The data was needed to calculate their energy intake and to assess eating pattern in order to plan a diet that emphasizes their preferences. Then both socio-demographic and dietary recall data were analyzed using statistical products and service solution (SPSS) program version 23.0. Descriptive analysis, which includes the mean, percentage and standard deviation, was used to find the average with its standard deviation. All the food items in the food list prepared were analyzed using Nutritionist Pro™ software and market survey to determine the nutrient content i.e. energy, macronutrients and most micronutrients. Lastly, Excel Solver was utilized to produce the linear programming model.

Before running the program, the details of each macronutrient and micronutrient of food items, price per serving size, were filled in Microsoft Excel. The next step was setting up the constraints in the model such as upper bound (UL) and lower bound (LB) for energy, macronutrients and micronutrients. Excel Solver was later used to determine the optimal foods portion size that met all the dietary and nutrient recommendation of WCRF/AICR 2007 [20], MDG 2010 [21] and RNI 2017 [19] with the lowest possible cost. From the suggested foods portion, a daily balanced menu was later planned The optimization model will be repeated several times to produce two more suggested palatable menus with the lowest possible costs.

### Linear programming model development

Cancer prevention diet models with the lowest cost were planned. The formulation for Linear Programming is as follows:

$$ {\displaystyle \begin{array}{l}\operatorname{Minimize}:z=\sum {c}_j{x}_j\\ {}\mathrm{Subject}\ \mathrm{to}:{b}_i\le \sum {a}_{ij}{x}_j\le {b}_i\ \mathrm{and}\ {x}_j\ge 0\end{array}} $$

The portion size of food item *j* is represented as *x*_*j*_*; a*_*ij*_ denotes the amount of nutrient *i* in one portion of food item *j*; *c*_*j*_ was the cost of a portion of food item *j*; *b*_*i*_ denotes the largest or smallest acceptable quantity of nutrient *i*. The constraints in the model for this study were WCRF/AICR 2007 [20], MDG 2010 [21] and RNI 2017 [19]. Palatability constraints were also included to ensure that the suggested menus were suited to the subjects’ common food pattern.

In this study, the cost of food items (*z*) is the objective function that we want to minimize. Subjects’ energy needs were calculated before the linear programming program started. The ideal energy of the subject was calculated using the Mifflin St-jeor Formula [22]. The minimum and maximum value of macronutrient and micronutrient were set based on WCRF/AICR 2007 [20], MDG 2010 [21] and RNI 2017 [19]. Choosing food items from the dietary recall of the subjects and avoiding the repetition or large portions of certain foods were also considered to ensure the palatability of the menu.

## Results

### Socio-demographic data

A total of 100 subjects from staff and students of participated in this study, with the females constitute the majority (65%) and the remaining balance of 35% was male. The mean age of female subjects was 24 ± 5.5 years, where more than 50% of female were between the ages 19 to 29 years. While, the mean age for male subjects was 25 ± 6 years, where more than 59% were between 19 to 29 years old. More than two-third (79%) was single and the rest (21%) were married. Based on the results, all of the subjects completed at least secondary schooling while 20% have completed a bachelor’s degree or higher.

### Anthropometric measurements

Anthropometric measurements were taken from the subjects. After the weight and height of the subjects were obtained, Body Mass Index (BMI) was calculated for each subject. Majority of the study population (48%) had normal BMIs between 18.5 kg/m^2^ and 24.9 kg/m^2^, which were almost similar to the prevalence of normal body weight of healthy Malaysian adults of 45.6% (National Health Morbidity Survey 2015) [23]. On the other hand, 33% of the subjects had BMIs between 25 kg/m^2^ and 29.9 kg/m^2^, thus belonging to the overweight classification. Prevalence of overweight for healthy Malaysian adults was 30%, which is almost similar to the subjects (National Health Morbidity Survey 2015) [23]. The remaining 19% were classified as obese.

### Dietary intake

All the macronutrients average intake of the subjects was met as shown in Table 1. Similarly, according to MANS 2008, the proportions of calories derived from macronutrients were within the recommendations for a healthy diet, while intake of micronutrients such as iron, calcium and vitamin A was about 50% of RNI particularly in women [24].

**Table 1:** The average energy/macronutrients/micronutrients intake with the recommendations and percentage of subjects achieving recommendations

Energy/Macronutrient / Micronutrient	Average Intake ± SP	Percentage of Intake from Energy	Recommendation	% Of subjects achieving Recommendation
Energy (kcal)	1580 ± 300			70.5
Protein (g)	56 ± 18.5	14.7% ± 4.6%	10–20%^3^	78
Carbohydrates (g)	203 ± 45.8	53.5% ± 11.2%	50–65%^3^	66
Fat (g)	48 ± 17.09	28.5% ± 9.5	25–30%^3^	59
Cholesterol (mg)	184 ± 91	–	< 300 mg/day^3^	89
Saturated Fat (g)	15 ± 6.8	9% ± 1%	< 10%^1^	79
Monounsaturated (g)	11 ± 5	6.5% ± 2.5%	12–15%^3^	2
Polyunsaturated (g)	9 ± 4	5.3% ± 2.5%	3–7%^3^	33
Fibre (g)	7.5 ± 4	–	25–30^1^	0
Sodium (mg)	2585 ± 544	–	< 2000^1^	20
Potassium	1181 ± 483	–	≥ 4700^3^	0
Vitamin A	954 ± 812	–	600–3000^3^	80
Beta-Carotene	1061 ± 813	–	2500^1^	6
Vitamin E (mg)	4 ± 2.3	–	7.5–1000^3^	10
Vitamin K (μg)	32 ± 20	–	55–1000^3^	15
Vitamin C (mg)	36 ± 25	–	70–2000^3^	8
Vitamin B12 (μg)	2 ± 1.5	–	≥ 4^3^	20
Folate (μg)	124 ± 76.6	–	400–1000^3^	2
Iron (mg)	13 ± 5.2	–	29–45^3^	0
Calcium	418 ± 226	–	1000–2500^3^	0
Thiamine (mg)	1 ± 0.2	–	≥1.1^3^	33
Riboflavin (mg)	1 ± 0.9	–	≥ 1.1^3^	45
Niacin (mg)	11 ± 4	–	14–35^3^	26
Zinc (mg)	5.5 ± 1.61	–	4.7–35^3^	30
Magnesium (mg)	124 ± 70.8	–	320–500^3^	0
Copper (mg)	0.6 ± 0.5	–	0.9–2^3^	10
Selenium (μg)	35 ± 18.4	–	24–400^3^	66
Phosphorus (mg)	822 ± 322	–	700–3000^3^	67
Sugar (g)	51 ± 19.8	–	< 10^3^ (50 g)	44

Source: ^1^WCRF/AICR 2007, ^2^MDG 2010, ^3^RNI 2017

Fiber intake was only 7.5 ± 4.05 g, thus failing to meet the prescribed recommendations (Table 1). Only 30% of the subjects meet the recommendations for fiber. The average intake of non-starchy vegetables and fruits were below the recommended amount of WCRF/AICR 2007 (2.4 versus 5 serving per day). In addition, the average intake of unprocessed grains and legumes were also below the recommendation (0.7 versus 3 serving). Malaysian Adult Nutrition Survey (MANS) 2014 [25] revealed that Malaysian adults on average do not consume sufficient fruits and vegetable in terms of frequency and amount, therefore does not achieve the recommended intake of fibers and other micronutrients. In this study, no subject met the requirement for iron and folic acid. Other micronutrients intakes such as calcium, vitamin B3, B12, vitamin C, vitamin E, vitamins K were also poor. Similarly, the MANS 2014 [25] also reported that the intake of micronutrients in relation to RNI could be described as low particularly for calcium and vitamin C intake. Healthy Eating Index for Malaysians showed that only a small percentage of Malaysian met dietary requirements and found that majority of the respondents (80.7%) were at risk of poor diet quality [26]. As for zinc, selenium and phosphorous, all subjects achieved the recommended requirements set by the RNI 2017. While for potassium, the average intake of a subject was 1181 ± 480 mg, and none of the subjects met the recommendation requirement of the RNI 2017, which is 4700 mg. The average intake for sugar and sodium were (51 ± 19.8 g) and (2585 ± 544 g) respectively, which exceeds the recommendations.

The average intake of red and processed meat is less than 300 g per week, meeting the recommendations of WCRF/AICRF 2007. However, the intake of processed food and salt was higher than the recommended amount. Likewise, MANS 2014 [25] showed that consumption of “processed foods” added with salt and condiments had increased and appeared among the top ten most consumed foods by Malaysian adults. Red meat consumption is associated with the formation of N-nitroso compounds. This increases the level of nitrogenous residues in the colon and is associated with the formation of DNA adducts in colon cells. High intake of red meat may result in more absorption of haem iron, greater oxidative stress and potential for DNA damage. Beside, red meat is high in animal fat and is energy dense food. All these factors contribute for considering red and processed meat as a cause of colorectal cancer [27].

### Development of cancer prevention model and menu with minimum cost

Linear programming has been used to formulate nutritionally optimal dietary patterns, to examine the relationship between diet cost and diet quality in Western countries and to develop food-based dietary guidelines in developing countries where residents need to achieve nutritional requirement with their limited income of diet [28]. Similarly, a Malaysian study done by Rajikan et al. developed a healthy and palatable diet for low income women at the minimum cost based on Malaysian Dietary Guidelines 2010 and Recommended Nutrient Intake 2005 via linear programming [29]. Optimization models provide an elegant mathematical solution that can help to determine that a set of dietary guidelines is achieved by Malaysian population subgroups. There were three models produced by linear programming. Table 2 shows all nutrients constrains and the food groups of the three different models produced by LP based on the dietary guidelines of WCRF/AICR 2007, MDG 2010 and RNI 2017. The three models produced, fulfilled the upper and the lower limits of the constrains including macronutrient and micronutrient recommendations set by WCRF/AICR 2007 and RNI 2017, the serving size of the food groups based on WCRF/AICR 2007 and MDG 2010. The palatability factor was also considered by including servings from vegetable oil and palm oil.

**Table 2:** Comparison of the three models produced with WCRF/AICR, MDG, RNI and palatability constrains

Constrain	LB	UB	Model I	Model II	Model III
WCRF/AICR 2007					
Non-Starchy Vegetables and fruits (serving/day)	5	–	13	14.5	11.5
Cooked Vegetables	–	–	6	3.5	6
Salad	–	–	1	2	–
Spices	–	–	4	6	3.5
Fruits	–	–	2	3	2
Red and Processed meat (serving/week)	–	3.5	0	0	0
Unprocessed grains and legumes (serving/day)	1	–	2	2	5
Fibre (g)	25	35	32.3	26	26.3
Sodium (g)	500	2000	563	1631	1853
MDG 2010					
Cereals and Grains (serving)	6	8	6.5	6	6
Meat/Poultry (serving)	1	2	1	1	2
Fish (serving)	1	3	1	1	2.5
Legumes (serving)	0.5	1	1.5	1	1
Palatability					
Vegetable oil (serving)	1	2		1	
Palm Oil (serving)	1	2		2	2
RNI 2017					
Energy (kcal)	1600	2000	1802	1685	1889
Protein (g)	42	100	69.5	58	102
Carbohydrates (g)	212	300	224	218	236
Fat (g)	38	68	62	59	56
Cholesterol (mg)	60	200	135.7	116	217
Saturated Fat (g)	10	22	16.5	10.5	19.9
Monounsaturated (g)	22	33	23	19.8	23.6
Polyunsaturated (g)	10	15	15	13.7	13.5
Potassium (mg)	4700	10,000	4729	4696	4704
Vitamin A (RE)	600	3000	4826	1913	1409
Beta-Carotene	2500	17,000	10,430	10,516	6365
Vitamin E (mg)	7.5	1000	12	10.2	13.2
Vitamin K (μg)	55	1000	55	71.6	99.7
Vitamin C (mg)	70	1000	549	233	274
Vitamin B12 (μg)	4	23	11.5	7.3	6.6
Folate (μg)	400	1000	413	450	475
Calcium (mg)	1000	2500	1000	1143	1113
Iron (mg)	29	45	29.5	30	29
Thiamine (mg)	1.1	500	10	2.2	1.8
Riboflavin (mg)	1.1	25	3.4	2.3	3.2
Niacin (mg)	14	35	32	29	28.5
Zinc (mg)	4.7	35	6.1	10.6	17.7
Magnesium (mg)	320	500	227	342	298
Copper (mg)	0.9	2	1.4	1.1	1.5
Selenium (μg)	24	400	47	43	23
Phosphorus (mg)	700	4000	1370	1507	1608
Sugar (g)	0	50	47	48	45

Looking at the three LP models as shown in Table 2, iron, potassium and calcium only reached the lower limit of the constraint values. However, other nutrients such as carbohydrate (CHO), fat, vitamin A and fiber reached the upper limit of the maximum acceptable value of constraints.

The food list selected comprised mainly on fruits and vegetables with the highest serving, as complex mixture of phytochemicals present in whole vegetables and fruits may have additive and synergistic effects responsible for anti-cancer activities [6]. Beside, these food items are low-energy density and high in fibers, which will provide sufficient fibers to meet the recommendations of 25-g fibers by WCRF/AICR and meeting the new recommendation of RNI 2017 for potassium.

From the suggested food list of the models, it is understood that each model consisted of at least two servings of whole and unprocessed grains such as brown rice, oat, lentils, and whole meal bread, thus ensuring high fiber and nutrient contents. The food list for each model also provides at least two servings of fruits and more than nine servings of vegetables, although it resulted in slight variation of the existing diets. In consequence, an optimal cancer prevention menu is developed based on a model that was produced using a linear programming method where it meets the requirements of constraints based on the dietary guidelines of WCRF/AICR 2007, MDG 2010, and RNI 2017.

Table 3 shows the three menus produced, according to raw food items at the lowest possible cost based on WCRF/AICR, MDG, RNI and palatability constraints by using LP. The production of every menu is different from another as it follows the list of food ingredients selected according to the LP models. For each model, every list of ingredients included in the model will have a slight difference by removing food items that have been selected in the previous menus or placing limits on the same food from a model to the next model so that quantities are different or not selected by the next model. Therefore, the price is expected to increase from menu 1 to menu 3 as the models have stricter requirement and the cheapest nutritionally dense foods have been chosen in the previous model. The development of these menus had different energy levels within the lower and the upper limit set for kcal (1600–2000 kcal). Model 1 is 1802 kcal with the lowest price RM 7.8 (USD 1.97), model 2 is 1680 with RM9.2 (USD 2.35) and model 3 is 1889 Kcal with the highest price RM 9.7(USD 2.48).

**Table 3:** Developments of three cancer prevention menus with minimal cost

Meal	Menu 1	Menu 2	Menu 3
Breakfast	Fried Kuey Teow (1 cup) Papaya (1 slice)	Laksa Penang with sardine (1 ½ cup) Pineapple (1 cup)	Hailam Noodle with chicken (1 cup) Banana (1 piece)
Morning snack	Bubur Kacang Hijau dan Keledek with sweet potatoes (1 ½ cup)	Oat cookies (2 pieces)	Cream Crackers (3 pieces) Coffee plain (1 cup)
Lunch	Steamed Brown Rice (3scoops) Fried Indian Mackerel (1 medium piece) Spinach Soup with tomato (1cup)	Steamed Basmati Rice (3 scoops) Soy Sauce Chicken (1 medium piece) Ulam Raja (1 cup) Steamed cabbage (1 cup)	Basmati Rice (3scoops) Grilled Catfish + Air Asam (medium piece) Sambal Lady Finger (1 cup)
Afternoon snack	Capati (1 piece) Dhal Gravy with turmeric and carrots (1 ½ cup)	Banana Oat Smoothie with milk (1 cup)	Peanut Butter Sandwich (1 sandwich) Chocolate Drink (1 cup)
Dinner	Steamed Brown Rice (3 scoops) Grilled Chicken Breast (2 pieces) Fried Pucuk Paku (1 cup) Guava (1 piece)	Fried Rice with sardine (3 scoops) Ulam Raja (1 cup) Stir fried Red spinach Orange/carrot juice (1 cup)	Basmati rice (3 scoops) Masak Lemak with tempeh and vegetables (1 cup) Mango (1 piece)
Food Cost per day (RM)	RM 7.8	RM 9.2	RM 9.7

## Discussion

Diets that give more emphasis to those plant foods that are high in nutrients, high in dietary fiber, and low in energy density, (non- starchy vegetables & fruits) probably protect against some cancers such as: mouth, pharynx, esophagus, stomach colorectal, lung, pancreas and prostate [20]. These foods are high in antioxidants (carotenoids, beta-carotene, lycopene and Allium) such as, pink sweet potatoes, papaya, tomato, onions, garlic, mango, carrots and fiber, which are low in energy density, and so, promote healthy weight.

In addition, we can observe that the menu also emphasizes on the intake of cruciferous vegetables, such as broccoli, mustard leaves, cabbage and cauliflower which are associated with the reduction in the risk of several types of cancer [30]. The traditional food tempeh, which is rich in phytoestrogens, is also included in the menu as seen in menu 3, as it is found to exhibit a plethora of different anti-cancer effects, including inhibiting proliferation [31]. Developing cancer prevention diet requires little modification from the existing diet mainly by increasing the vegetables and fruit serving.

The recommended menu used only a dash of salt in seasoning the dishes and this causes the resulting menu to have a low sodium amount of less than 2000 mg. Studies showed that salt and salt-preserved foods are probably a cause of stomach cancer [32]. The other alternatives are to use natural flavoring to replace salt are turmeric, onions, garlic, chili and mustard leaves that contain lower sodium content.

The menu also restricted the use of added sugar and the intake of sugary drinks, except for sugar that is naturally found in fruits and vegetables. Instead, healthy high antioxidant drinks were suggested such as carrot and orange juice. Furthermore, it is evident that the diet models do not include any processed meat, fast food, or sugary drinks; where lean proteins were the only protein source.

Looking at the fat content in the three models, we can see that it emphasizes on less saturated fats and trans-fat by reducing the consumption of fat, which is mainly achieved by appropriate cooking methods. Based on the menu that has been set up, almost all models use a minimal of 3 tablespoons of oil. Therefore, the menu is provided with many ways of cooking such as steaming, baking or grilling. In a study conducted by Asmaa et al. [33], the use of healthy cooking methods such as steaming can reduce fat content in food.

However, there were few limitations in this study. The subjects in this study may have not been representative because they were not randomly sampled from the general Malaysian population, rather, they were only limited to a local university staff and students. A larger number of subjects were from different economic and social background and thus more lists of food items should be included in the model to increase the variety of food choices in future studies.

## Conclusion

In general, the use of linear programming is a very effective tool in producing a balanced diet and can easily interpret dietary recommendations into a nutritional model that is based on local market prices. It formulated the current guidelines for cancer prevention by creating a balanced and optimal diet for cancer prevention at minimum cost with more specific details and accuracy. In addition, because this research focuses on the specific nutrients needed at minimal cost, the menus produced are ideal for people who want to maintain healthy eating habits but experience financial difficulties.

## Abbreviations

AICR: American Institute for Cancer Research
BMI: Body Mass Index
DNA: Deoxyribonucleic Acid
DRI: Dietary Recommended Intake
KPDNKK: Ministry of Domestic Trade, Cooperative, and Consumerism
LB: Lower bound
LP: Linear Programming
MANS: Malaysian Adult Nutrition Survey
MDG: Malaysian Dietary Guidelines
NHMS: National Health Morbidity Survey
RNI: Recommended Nutrient Intake
UKMREC: Universiti Kebangsaan Malaysia (National University of Malaysia) Medical Research Ethics Committee
UL: Upper bound
WCRF: World Cancer Research Fund
